# Guidelines for Integrating actionable A-SMART Learning Outcomes into the Backward Design Process

**DOI:** 10.12688/mep.20606.1

**Published:** 2024-10-22

**Authors:** Carlos Kiyan Tsunami, Aquiles Rodrigo Henríquez-Trujillo, Karen Ferreira-Meyers, Ziyanda Mwanda, Jyostna Rimal, Jamine Pozu-Franco, Thérèse Delvaux, Deogratias Katsuva Sibongwere, Héctor Javier Montalvo Navarrete, Anuttama Dasgupta, Jean Michel Kolie, Gradi Luakanda-Ndelemo, Claude T. Semevo, Sotheara Heng Heng, Susan Dierickx, Diljtih Kannan, Harish Hn, Luis Fucay Guin, Kranthi Vysyaraju, Maria Zolfo

**Affiliations:** 1 Institute of Tropical Medicine, Antwerp, Antwerp, 2000, Belgium; 2 University of Eswatini, Kwaluseni, Swaziland; 3 University of the Free State, Bloemfontein, South Africa; 4 School of Public Health, University of Western Cape, Belville, Western Cape, 7535, South Africa; 5 BP Koirala Institute of Health Sciences, Dharan, Province No. 1, Nepal; 6 Universidad Peruana Cayetano Heredia, Lima District, Lima Region, Peru; 7 Pontifical Catholic University of Ecuador Faculty of Medicine, Quito, Pichincha, Ecuador; 8 Indian Institute for Human Settlements, Bengaluru, Karnataka, India; 9 Le Centre National de Formation et de Recherche en Sante Rurale, Maferinyah, Guinea; 10 L’Institut National de Recherche Biomédicale, Kinshasa, Democratic Republic of the Congo; 11 Centre de recherche en reproduction humaine et en démographie, Cotonou, Benin; 12 National Institute of Public Health, Phnom Penh, Phnom Penh, Cambodia; 13 Indian Institute of Management Bangalore, Bengaluru, Karnataka, India; 14 Institute of Public Health, Bangalore, Karnataka, 560070, India

**Keywords:** A-SMART Learning Outcomes, AI, Backward Design, Bloom's Taxonomy, Instructional Design, Student-centered Learning, Real-World Applications

## Abstract

**Background:**

Learning outcomes are essential in education, guiding both educators and learners towards desired knowledge, skills, and competencies. The backward design process offers a structured approach to curriculum planning, but its integration with actionable, SMART (Specific, Measurable, Achievable, Relevant, Time-bound) learning outcomes needs further exploration.

**Goal:**

This guide aims to introduce the concept of "A-SMART" learning outcomes and demonstrate their integration into the backward design process, focusing on outcomes that begin with action verbs.

**Methods:**

The guide outlines a three-stage curriculum planning approach: (i) define desired results, (ii) determine acceptable evidence of learning, and (iii) plan learning activities. It emphasizes the importance of starting with action verbs in formulating learning outcomes, aligning with Stage 1 of backward design and facilitating the transition to Stage 2 (assessment development).

**Results:**

By following this guide, educators will acquire tools to develop effective "A-SMART" learning outcomes. This approach immediately advances to Stage 2 of backward design, improving educational practices and ensuring alignment with assessment methods. The guide provides strategies for formulating outcomes that are Action-oriented, Specific, Measurable, Achievable, Relevant, and Time-based.

**Conclusions:**

The integration of A-SMART learning outcomes into the backward design process offers a more cohesive and effective educational framework. This approach enhances clarity for learners, provides guidance for instructors, enables more effective assessments, and improves overall learning experiences. The guide also addresses potential challenges in formulating A-SMART outcomes and suggests solutions, including the use of AI tools for inspiration and critical review.

## Introduction

Learning outcomes are defined as a list of statements describing the skills and knowledge that course participants should acquire by the end of a course ^1^. Learning outcomes can be technical (skill-based, what participants can do) or academic (knowledge-based, what participants should know), depending on the targeted participants, their learning needs, and the nature of the learning activity (course, training, or seminar). Learning outcomes are crucial for providing clear targets for educators and learners. This guide offers practical strategies for formulating effective A-SMART learning outcomes as the first stage of backward design–a curriculum planning approach that starts with the end goals and works backward to develop the curriculum. This method enhances educational practices and ensures alignment with assessment methods ^2^. However, the learning outcomes should not be formulated in a vacuum. They should reflect on the needs or gaps of the target audience. Learning outcomes should follow needs or situational assessments, in which the targeted audience’s learning needs or gaps are explored in detail. This activity ensured that the course was student-centered. Relevant resources and literature have shown that starting with a needs assessment can improve a course’s impact and participant satisfaction ^3^. As a starting point, the needs assessment results form the basis of the learning outcomes.

While many resources discuss the formulation of SMART outcomes or objectives ^4– 8^, this guide emphasizes starting with an action verb (the "A" in A-SMART). This approach immediately aligns with Stage 1 of the backward design ^9^. The proposed approach clarifies the desired outcome and instantly suggests considering Stage 2 (how to assess it) ^2, 9, 10^, thereby creating a more integrated and cohesive educational framework oriented to evaluate the level of achievement.

While many resources discuss the formulation of SMART outcomes or objectives ^4– 8^ this guide emphasizes the significance of integrating A-SMART learning outcomes into the backward design process. This approach not only clarifies desired outcomes, but also facilitates the alignment of assessments and instructional strategies, creating a more cohesive and effective educational framework.

## Why use A-SMART learning outcomes?

A-SMART learning outcomes are preferred over traditional objectives because they focus on a student-centered perspective ^10^ by clearly defining what learners are expected to achieve. By starting with an action verb (A), this approach aligns with the first stage of the backward design ^11^, prompting immediate consideration of how to assess the expected outcome ^9, 12^. The A-SMART framework defines effective learning outcomes as

1. **Action-oriented:** outcomes begin with an appropriate action verb, aligning with Stage 2 of the backward design, to consider how to measure immediately ^11^.
2. **Specific:** Outcomes define the expected learning clearly, avoiding ambiguous language ^4, 7^.
3. **Measurable:** Outcomes can be measured using clear criteria ^2, 11^.
4. **Achievable:** Outcomes consider available resources and realistic expectations ^2, 11^.
5. **Relevant:** Outcomes are meaningful and applicable to real-world settings ^2, 11^.
6. **Time-based:** Outcomes allocate sufficient time for achievement ^2, 11^.

## Backward design: aligning A-SMART outcomes, assessments, and learning activities

Backward design is a three-stage approach that ensures the alignment of learning outcomes, assessments, and learning activities ^12^. The first stage involves formulating clear and measurable A-SMART learning outcomes that are crucial for the entire process ^12^. The second stage focused on determining appropriate assessment methods that provide strong evidence of student achievement ^12^. The third stage involves designing the learning activities and their sequence to prepare students for the assessment, and determining what content and experiences should be provided to learners.

## Reflect on real-world applications

Before formulating outcomes, consider the real-world contexts in which learners apply their knowledge, skills ^2, 6^ or attitudes (if you can objectively and accurately measure them with evidence). By considering these, instructional designers ensure that outcomes are not just theoretically sound, but also practically relevant.

For example, in a surgery course during a pandemic where face-to-face meetings are not possible, reflecting real-world applications could involve teaching suturing techniques remotely. A relevant learning outcome for students would be:

“Demonstrate proper suturing technique on a simulated wound using a home suture kit, achieving correct knot tying and wound closure as evaluated through a video submission, while adhering to sterile technique principles” ^6^.

## Formulating A-SMART learning outcomes

1. **Start the learning outcome with an action verb:** Use verbs that clearly define the expected skills or knowledge ^2, 7^.

2. **Focus on specific and observable actions:** Replace vague verbs with specific actions ^6^. Example: Instead of "understand", use "describe" or "explain".

3. **Avoid ambiguous language:** Remove subjective or hard-to-measure words ^6, 7^. Example: Replace "know" with "identify" or "list".

4. **Single Action Focus:** Each outcome should focus on one specific action ^7^. Example: "Prepare a fluffy, well-cooked omelet with a variety of fillings’".

5. **Use Measurable Terms:** Start each outcome with a measurable verb and clearly describe the expected action ^7, 13^. For example: "List the steps and ingredients required to prepare a fluffy, well-cooked omelet with at least three different fillings”.

6. **Specify contextual conditions:** Describe the conditions under which the action will occur, particularly in specialized fields ^2, 10^. For example, "prepare a fluffy, well-cooked omelet with a variety of fillings of their choice, using a gas stove, while demonstrating proper cooking techniques, such as controlling heat levels and monitoring cooking times, as well as adhering to food safety practices, including proper handling of raw eggs, cleaning surfaces, and maintaining appropriate temperature control’".

7. **Avoid describing instructional activities:** focus on what the learner should be able to do after instruction, not on instructional methods. Here is a bad example: "practice preparing a fluffy, well-cooked omelet with a variety of fillings of their choice using a gas stove. The instructor will guide them by controlling heat levels, monitoring cooking times, and teaching them proper food safety practices, such as handling raw eggs safely, cleaning surfaces, and maintaining appropriate temperature control **after attending a lecture on omelet-making techniques and watching cooking demonstrations**".

8. **Use Bloom's Taxonomy Verbs:** Select appropriate verbs from Bloom's taxonomy to ensure clarity and measurability ^6, 14^. Examples include Create, Evaluate, Analyze, Apply, Understand, Remember.

## Exploratory learning outcomes drafted with the aid of Artificial Intelligence (AI)

AI can be used to help create and review learning outcomes ^15^. To review or formulate learning outcomes, consider using the following AI prompts:

(Copy and paste it into a text editor and modify it accordingly).

“I have the following learning outcome that the target audience [insert the profile] should be for a [insert beginner/intermediate/advanced level]: [Insert your learning outcome here]. Could you evaluate this learning outcome using the A-SMART criteria, and provide feedback on how it can be improved to meet these criteria? Additionally, please explain how this learning outcome can be applied in a real-world context so that students can understand its practical relevance. Your responses include the following.

1. An assessment of whether the learning outcome meets the A-SMART criteria (Action-oriented, Specific, Measurable, Achievable, Relevant, and Time-based), with specific comments or suggestions for improvement if any requirements are not met.

2. A clear explanation of how this learning outcome can be applied in a real-world setting or workplace scenario, providing examples or use cases to illustrate its practical relevance.

3. If necessary, a revised version of the learning outcome addresses any identified issues and meets all A-SMART criteria while maintaining its real-world applicability.

Please ensure your feedback is detailed, constructive, and easy for educators and students to understand”.

## Example of an A-SMART learning outcome

"Perform a simple interrupted suture on a simulated skin wound model, demonstrating proper instrument handling, correct suture placement, appropriate knot tying technique, and maintenance of sterile field, completing the procedure within 10 minutes and achieving a wound closure score of at least 8 out of 10 on the standardized assessment rubric ^6^.

## Benefits of A-SMART learning outcomes into backward design

1. **Clarity for Learners:** Students understand what is expected of them, fostering motivation and engagement ^2, 10^.
2. **Guidance for Instructors:** Educators can align teaching strategies, assessments, and learning activities with clearly defined outcomes ^9, 12^.
3. **Enhanced Assessment:** Clear outcomes enable the creation of more effective and aligned assessments that accurately measure student learning ^2, 12^.
4. **Improved Learning Experience:** Students are more likely to achieve desired results when the outcomes are clear and achievable ^13^.

## Challenges and solutions in formulating A-SMART learning outcomes

1. **Challenge:** Crafting measurable and specific outcomes can be time-consuming.
*Solution:* Using proper prompts, AI can inspire A-SMART-compliant learning outcomes ^15^.
  While AI tools can improve efficiency and provide starting points to consider when developing learning outcomes, you are encouraged to critically review the responses, looking out for context/cultural appropriateness to avoid perpetuating bias or overlooking the real needs of your targeted participants.
2. **Challenge:** Balancing specificity and generality to ensure outcomes are achievable but not overly prescriptive.
*Solution:* Pilot the outcomes with a small group of students to ensure that they are clear and achievable ^16^.

## Conclusion

These guidelines provide a structured approach to formulating A-SMART learning outcomes to enhance the effectiveness of educational practices. Educators can create clear and achievable targets that align with assessment practices and improve students’ learning experiences.

Planning results that consider real-world contexts favor the application of students’ knowledge and attitudes.

## Consent

This study is a theoretical analysis and does not involve human participants, animals, or any experimental procedures. Therefore, no consent was required for this research. All information and conclusions presented are based on previously published literature, which is appropriately cited within the manuscript.

## Data Availability

This study did not generate or analyze any datasets. The theoretical analysis presented in this manuscript is based solely on previously published literature, which is cited within the text. No software was used in conducting this research.

## Reporting guidelines

This manuscript adheres to the reporting guidelines for theoretical analyses and educational frameworks. The development of A-SMART learning outcomes was guided by established principles of backward design and instructional design methodologies. The recommendations made in this guide are based on a synthesis of existing literature and best practices in education, ensuring that the proposed methods align with recognized educational standards.

No specific reporting guidelines were required for this theoretical analysis as it does not involve empirical research or data collection.
