# Supplementary material for: Guidelines for Integrating actionable A-SMART Learning Outcomes into the Backward Design Process
Source: MedEdPublish (2016). 2024 Oct 22;14:242. [Version 1] doi: 10.12688/mep.20606.1 (PMC11589412; doi:10.12688/mep.20606.1)
Supplement: Peer review history

# Reviewer report: Reviewer response for version 1

**DOI:** 10.21956/mep.22058.r39594

**Published:** 2024-11-25

**Authors:** Oscar Mauricio Jerez Yañez

**Affiliations:** 1 Universidad de Chile, Santiago, Chile

The article presents a clear and practical guide, supported by solid literature, for integrating A-SMART learning outcomes into the backward design process. Its strengths lie in the well-structured framework and comprehensive theoretical foundation. However, there are opportunities to enhance its impact and applicability. These include diversifying examples to encompass a wider range of disciplines, such as humanities and social sciences, to demonstrate the framework's versatility. Additionally, expanding the discussion on specific AI tools to aid in the formulation of learning outcomes and providing a detailed case study that illustrates the application of the framework across the three stages of backward design would further strengthen its practical value and accessibility for educators in various fields.

If evidence from practice is presented, are all the underlying source data available to ensure full reproducibility?

Not applicable

Is the topic of the practical tips discussed accurately in the context of the current literature

Yes

Are all factual statements correct and adequately supported by citations?

Yes

Are the conclusions drawn balanced and justified on the basis of the presented arguments?

Yes

Are arguments sufficiently supported by evidence from the published literature and/or the authors’ practice?

Yes

Reviewer Expertise:

Medical Education

I confirm that I have read this submission and believe that I have an appropriate level of expertise to confirm that it is of an acceptable scientific standard.

# Reviewer report: Reviewer response for version 1

**DOI:** 10.21956/mep.22058.r39590

**Published:** 2024-10-30

**Authors:** Shahid Rafiq

**Affiliations:** 1 Emerson University Multan, Multan, Punjab, Pakistan

The article presents a valuable framework for incorporating A-SMART (Action-oriented, Specific, Measurable, Achievable, Relevant, Time-based) learning outcomes into the Backward Design process to enhance educational clarity, instructional alignment, and assessment precision. While the core concepts are well-structured and clearly articulated, the article would benefit from specific enhancements to broaden its applicability and strengthen its scientific rigor. First, while the explanation of A-SMART outcomes is effective, additional examples across diverse fields such as the humanities and social sciences would illustrate its versatility beyond skill-based contexts and make the guidance relevant to a wider audience. Additionally, the integration of A-SMART outcomes within Backward Design could be further clarified with a scenario-based example that demonstrates the practical impact of such outcomes across the three stages of the model. For instance, illustrating how a business course outcome might guide both assessment design and instructional activities would provide concrete insight into the Backward Design process in action. Finally, the discussion on challenges, particularly regarding the time investment for crafting specific outcomes, could be enhanced by expanding on AI tools as a solution. Providing specific examples of AI prompts or tools for outcome formulation would offer educators actionable steps to streamline the process. These improvements broadening examples, adding a practical scenario, and detailing AI applications would make the article more comprehensive, accessible, and scientifically robust for educators across disciplines

If evidence from practice is presented, are all the underlying source data available to ensure full reproducibility?

Yes

Is the topic of the practical tips discussed accurately in the context of the current literature

Yes

Are all factual statements correct and adequately supported by citations?

Yes

Are the conclusions drawn balanced and justified on the basis of the presented arguments?

Yes

Are arguments sufficiently supported by evidence from the published literature and/or the authors’ practice?

Yes

Reviewer Expertise:

Educational Evaluation, Teacher Education, Teacher Performance, Teacher Teaching Practices. Flipped classroom , Blended Learning

I confirm that I have read this submission and believe that I have an appropriate level of expertise to confirm that it is of an acceptable scientific standard.
